# GABAergic System in Stress: Implications of GABAergic Neuron Subpopulations and the Gut-Vagus-Brain Pathway

**DOI:** 10.1155/2020/8858415

**Published:** 2020-08-01

**Authors:** Xueqin Hou, Cuiping Rong, Fugang Wang, Xiaoqian Liu, Yi Sun, Han-Ting Zhang

**Affiliations:** ^1^Institute of Pharmacology, Shandong First Medical University & Shandong Academy of Medical Sciences, Tai'an, Shandong 271016, China; ^2^The Second Clinical Medical College, Guangzhou University of Chinese Medicine, Guangzhou, Guangdong 510006, China; ^3^Departments of Neuroscience and Behavioral Medicine & Psychiatry, The Rockefeller Neurosciences Institute, West Virginia University Health Sciences Center, Morgantown, WV 26506, USA

## Abstract

Stress can cause a variety of central nervous system disorders, which are critically mediated by the *γ*-aminobutyric acid (GABA) system in various brain structures. GABAergic neurons have different subsets, some of which coexpress certain neuropeptides that can be found in the digestive system. Accumulating evidence demonstrates that the gut-brain axis, which is primarily regulated by the vagus nerve, is involved in stress, suggesting a communication between the “gut-vagus-brain” pathway and the GABAergic neuronal system. Here, we first summarize the evidence that the GABAergic system plays an essential role in stress responses. In addition, we review the effects of stress on different brain regions and GABAergic neuron subpopulations, including somatostatin, parvalbumin, ionotropic serotonin receptor 5-HT3a, cholecystokinin, neuropeptide Y, and vasoactive intestinal peptide, with regard to signaling events, behavioral changes, and pathobiology of neuropsychiatric diseases. Finally, we discuss the gut-brain bidirectional communications and the connection of the GABAergic system and the gut-vagus-brain pathway.

## 1. Introduction

Stress is associated with various effects and mental disorders. Responses to stress vary from diet alteration to movement and sleep changes. Acute stress, such as trauma, can lead to rapid emotional changes and even result in long-term mental impairments. For instance, posttraumatic stress disorder (PTSD), a typical mental disorder, is often accompanied by depression and anxiety [1]. Chronic stress exposure, such as life stress (interpersonal loss, physical danger, humiliation, entrapment, role change/disruption, etc.), also increases depressive response and anxiety, and even triggers suicide in extreme cases [2–5]. Both acute and chronic stress-induced mental problems are associated with the *γ*-aminobutyric acid (GABA) system [6–9]. Gene polymorphism analysis of healthy subjects indicates that the GABA(A)*α*6 receptor subunit gene (GABRA6) polymorphism is responsive to psychological stress [10]. Therefore, agents targeting the GABAergic system are used to regulate depression, anxiety, or fear [11–13]. Interestingly, growing evidence has shown that gut-brain signals influence emotional behaviors [14–16], and the gut-brain axis may be a possible target for treating stress-related disorders [17]. A recent review has summarized the psychophysiological effects of prebiotics and discussed the important roles of bacteria-gut-brain signals in psychobiotic activity [18]. In addition to the gut microbiome, neurotransmitters and neuropeptides are also involved in the gut-brain communications [19–21]. Some neurotransmitters and neuropeptides in the central nervous system (CNS) are involved in regulating the function of the digestive system [22–25]. Moreover, some neuropeptides are expressed in GABAergic neurons, which may be parts of the GABAergic system. In this review, we focus on how the GABAergic system impacts the gut-brain interaction in order to mediate stress-related disorders.

## 2. GABAergic Neuron Signaling and Stress

GABA is a major inhibitory neurotransmitter and is synthesized from the amino acid glutamate regulated by glutamate decarboxylases (GADs), including GAD1 and GAD2, whose genes encode GAD67 and GAD65 proteins, respectively [26]. GABAergic neurons are widely distributed in the CNS of mammals, and together with other GABA related factors, they compose the GABAergic system. The ventral medial prefrontal cortex (vmPFC) responds to the GABA reuptake inhibitor tiagabine [27] and is associated with fear responses and stress [28, 29], suggesting that the vmPFC GABAergic system plays a role in regulating stress-related emotion and responses. Additionally, many other GABAergic neuron-containing brain structures (such as the hippocampus and amygdala) and GABA-associated signaling are also involved in the stress regulation [30, 31].

GABAergic neurons coexpress various proteins or neuropeptides, such as somatostatin, parvalbumin, ionotropic serotonin receptor 5-HT3a (5-HT3aR), cholecystokinin, neuropeptide Y (NPY), vasoactive intestinal peptide, calbindin, and calretinin [32–34]. In addition, ~40% of GABAergic neurons are parvalbumin interneurons, ~30% are somatostatin interneurons, and ~30% are 5-HT3aR interneurons in the neocortex [35], which make up the three major subtypes of GABAergic neurons. Other proteins or neuropeptides are expressed in different subtypes of GABAergic neurons. For example, cholecystokinin and vasoactive intestinal peptide may express in 5HT3aR interneurons, and NPY is colocalized with somatostatin interneurons [35].

The changes in subpopulations of GABAergic neurons vary in different brain areas under stress [36, 37]. For example, long-term daily stress reduces the number of parvalbumin, calretinin, NPY, and somatostatin cells, but does not affect cholecystokinin and calbindin interneurons in the hippocampus [36]. Early life stress changes the structure and function of several brain regions, in addition to alterations of emotional behaviors and responses to stress in adults [38–40]. Exposure to long-term daily stress reduces calbindin neuron densities in the dorsolateral, medial, and ventral orbital cortex, but has no effect on cholecystokinin, NPY, parvalbumin, somatostatin, and calretinin neurons in any brain subregions in adult rats. Interestingly, enhanced density of cholecystokinin and NPY neurons in the ventral and lateral orbital cortices, respectively, is observed in stress-resilient rats, suggesting that cholecystokinin and NPY in the orbitofrontal cortex may be involved in stress resilience [41]. Taken together, these results suggest a complex GABAergic network change under stress. Also, it raises a question of how to control the GABAergic network in order to regulate stress-induced emotional behaviors. Of those coexpressing markers in GABAergic neurons, cholecystokinin, NPY, and vasoactive intestinal peptide are also known as gut-related modulators and involved in the regulation of energy. Subsequently, how do they connect to GABA signaling to play a role in the regulation of stress?

### 2.1. Somatostatin

Somatostatin is a chemical marker of GABAergic neurons [33]. Somatostatin deficit is a common pathological characteristic in neurological disorders with emotional changes. In patients with schizophrenia and bipolar disorder, somatostatin-immunoreactive neurons are decreased in the lateral amygdala, which may affect responses to fear and anxiety [42]. Mice deficient in somatostatin exhibits high behavioral emotionality, increased basal plasma corticosterone, and decreased GABA-synthesizing enzyme GAD67 gene expression [43], indicating that somatostatin influences the GABA signal and stress response. Upon 2 weeks of chronic mild stress in rats, somatostatin-2 receptors are significantly upregulated in the medial habenula, while the plasma somatostatin levels are also increased, suggesting that somatostatin and its receptors are involved in the stress response [44]. Longer duration (e.g., 7 weeks) of chronic mild stress in rats can cause decreases in consumption of sucrose solution and changes in somatostatin-2 receptors in response to antidepressant treatment [45]. Moreover, selective inactivation of the *γ*2 subunit gene of GABAA receptors in somatostatin-positive GABAergic interneurons (SSTCre:*γ*2(f/f) mice) mimic the behavioral effects of antidepressant and anxiolytic drugs, suggesting that sustained increases in GABAergic transmission produce antidepressant-like behavior by disinhibiting somatostatin-positive GABAergic interneurons [46].

### 2.2. Parvalbumin

Parvalbumin is another chemical marker of GABAergic neurons. Parvalbumin and somatostatin interneurons play distinct roles in the medial entorhinal cortex [47], a critical brain region associated with contextual memory [48]. Besides, parvalbumin- and somatostatin-expressing interneurons in the mPFC also have different activity patterns (weak and strong target-dependent delay-period activity), as well as distinct stimulation effects in spatial working memory. For instance, parvalbumin interneurons are strongly inhibited by reward, while only a subtype of somatostatin interneurons is inhibited [49]. Thus, parvalbumin and somatostatin interneurons may function in different ways. Selectively silencing parvalbumin, but not somatostatin, interneurons in the infralimbic cortex eliminates ventral hippocampal-mediated inhibition, while blocking infralimbic projectors reduces fear renewal [50], indicating that parvalbumin interneurons are involved in fear responses. Parvalbumin/GAD1 transgenic mice (silencing the GAD1) exhibit reduction of fear extinction, marked sensorimotor gating deficits, and elevated novelty-seeking [51]. Inhibition of parvalbumin interneurons disinhibits projection neurons from the prefrontal region and synchronizes their firing, resulting in fear [52]. After fear conditioning, parvalbumin interneurons show target- and region-selective plasticity in basolateral amygdala (BLA) subareas [53]. Together, parvalbumin interneurons regulate stress-induced fear, and fear affects the parvalbumin interneurons in return.

### 2.3. HT3aR

Among the serotonin (5-HT) receptors in mammals, the 5-HT3R is the only ligand-gated ion channel receptor for 5-HT. The 5-HT3aRs are found in cholecystokinin positive and vasoactive intestinal peptide positive GABAergic interneurons, and these 5-HT3aR-expressing vasoactive intestinal peptide/cholecystokinin interneurons receive serotonergic and cholinergic fast synaptic transmission [54]. Furthermore, coexpression of 5-HT3aR and central calbindin 1 cannabinoid receptors have been detected in GABAergic neurons in the anterior olfactory nucleus, the cerebral cortex, hippocampus, dentate gyrus, subiculum, entorhinal cortex, and amygdala [55, 56]. Interestingly, the activation of 5-HT3 receptors by serotonin causes GABA release, whereas stimulation of calbindin 1 receptors by cannabinoids inhibits GABA release, indicating opposing effects on GABA neurotransmission [55]. The amygdala has been known to be involved in the regulation of emotion. A moderate density of 5-HT3aR neurons are found in the amygdalar basolateral nuclear complex, and almost all 5-HT3aR neurons are GABA positive. Therefore, serotonin may activate 5-HT3 receptors in the 5-HT3aR positive GABAergic neurons in the amygdala and lead to GABA release, resulting in emotional changes under stress.

### 2.4. Cholecystokinin

Cholecystokinin is a peptide hormone produced by enteroendocrine cells of the small intestine and released into the blood. Cholecystokinin is also widely distributed throughout the CNS, with high levels in the limbic system. The sulfated octapeptide, cholecystokinin-8S, is the major biologically active form of cholecystokinin in the CNS [57]. Intraperitoneal injections of cholecystokinin-8 enhance c-Fos (an immediate-early gene) expression in the dorsal CA3 and dentate gyrus of the hippocampus [58], indicating that cholecystokinin-8 activates neurons in the hippocampus. In the dentate gyrus, the activation of presynaptic 5-HT1B receptors in cholecystokinin interneurons inhibits GABA release and further disinhibits parvalbumin interneurons, leading to reduction of the granule cells activity. Furthermore, the inhibition of cholecystokinin neurons exhibits antidepressant-like effects on behavior, similar to selective serotonin reuptake inhibitors [59]. Thus, the activation of cholecystokinin neurons affects GABA release and depressant-like behavior.

Cholecystokinin-4, another form of cholecystokinin, has been known to induce panic attacks. In a double-blind, placebo-controlled study, 26 of 30 subjects exhibited obvious panic responses when they were challenged with cholecystokinin-4 [60]. Subjects who were treated with anxiolytics alprazolam prior to the rechallenge of cholecystokinin-4 showed a significant reduction of the panic-related scale scores and reported symptoms, as well as lower adrenocorticotropic-hormone and cortisol release. Because slow GABABR-mediated inhibitory postsynaptic currents were recorded in most cholecystokinin interneurons [61], it is possible that cholecystokinin interacts with the GABAergic system. Systemic activation of cholecystokinin-GABA neurons by clozapine-N-oxide in triple transgenic cholecystokinin-GABA/hM3Dq mice, in which about 22% of GABAergic neurons in the hippocampus and 19% in the prefrontal cortex are cholecystokinin-GABA neurons, not only enhanced contextual fear conditioning/discrimination, social/object recognition, and puzzle box performance, but also enhanced anxiety in the elevated plus maze [62].

### 2.5. NPY

NPY is a peptide derived from the brain and sympathetic nerves and involved in various functions in both the peripheral and central nervous systems. In the periphery, NPY is mainly released from the sympathetic nerves and serves as a regulator of fat growth [63]. In the brain, it is produced in various regions (such as the hypothalamus and amygdala) and is implicated in multiple functions, including energy homeostasis, food intake, metabolism, and stress response [64–67]. Stress can increase NPY expression in the brain [67]. Also, stress response and emotion can be affected by human NPY expression, as lower haplotype-driven NPY expression is related to higher emotion-induced activation of the amygdala [68]. Thus, NPY is thought to have stress-relieving and anxiolytic properties [69]. Chronic unpredictable stress for 5 weeks has been shown to reduce GAD67 protein levels in the prefrontal cortex and hippocampus in rats, without changing GAD65 protein expression. Additionally, the protein and RNA levels of somatostatin and NPY are also decreased following stress exposure, suggesting these subsets of GABAergic neurons may be sensitive to chronic stress [70]. NPY is colocalized with somatostatin interneurons in the brain [35]. In the BLA, somatostatin interneurons express NPY2-receptors, some of which coexpress NPY; stimulating BLA NPY2-receptors reduces tonic GABA release onto local principal neurons [30]. A combination of stress and high-fat diet activates central amygdala NPY neurons, resulting in increased feeding and reduced energy expenditure [67].

### 2.6. Vasoactive Intestinal Peptide

Vasoactive intestinal peptide, a gut hormone regulating energy metabolism [71, 72], is produced in many tissues, such as the gut and the hypothalamic suprachiasmatic nucleus in the brain [71, 73]. In the CNS, neocortical vasoactive intestinal peptide positive neurons are one subpopulation of GABAergic interneurons [74]. Vasoactive intestinal peptide increases GABA release in the hippocampus without changing glutamate release. Concerted synaptic action of vasoactive intestinal peptide causes disinhibition of pyramidal cell dendrites and enhances GABAergic transmission [75]. The connections between different types of GABAergic neurons result in disinhibitory effects. For example, in the primary somatosensory cortex, most of the parvalbumin cells are innervated by vasoactive intestinal peptide neurons [76]. Therefore, neocortical vasoactive intestinal peptide positive GABAergic neurons send outputs onto other interneurons or principal neurons and display a disinhibitory effect [74]. Vasoactive intestinal peptide modulates hippocampal synaptic GABAergic transmission via activation of two vasoactive intestinal peptide receptors, i.e., VPAC1 and VPAC2 receptors, which, however, possess opposite effects on GABA release, as activation of VPAC1 or VPAC2 receptors inhibits or enhances GABA release, respectively [77]. Together, vasoactive intestinal peptide can affect GABAergic neurons, GABA level, and GAD expression [78, 79], which may further influence the stress-related behaviors.

## 3. Crosstalk between the GABAergic System and the Gut-Brain Pathway in Stress

### 3.1. The Vagus Nerve-Mediated Gut-Brain Pathway

The vagus nerve is an important neuronal component of the bidirectional communication of the gut-brain axis [80]. In addition to regulating the ingestive behavior, vagal afferent signaling has been implicated in the modulation of mood and affect, such as motivation and depression [81, 82]. Abdominal vagal afferents in rats display anhedonic behavior and increase behavioral despair [82]. It has been reported that disrupted vagal afferent signaling by subdiaphragmatic vagal deafferentation results in brain transcriptional changes in functional networks associated with schizophrenia, as well as dopamine alteration in the nucleus accumbens [83]. In another study, subdiaphragmatic vagal deafferentation rats exhibited a reduction in innate anxiety-like behavior assessed by open field test, elevated plus maze test, and food neophobia test, whereas their learning auditory-cued fear was increased [84]. Furthermore, these behavioral changes were related to the alterations of GABA and noradrenaline levels in the limbic system, without functional changes in the hypothalamus-pituitary-adrenal grand stress [84]. It suggests that vagal afferents may connect with the limbic system and affect the GABAergic system in the CNS. Selective ablation of gastrointestinal vagal sensory/afferent by saporin-based lesion impaired hippocampus-dependent behaviors in rats, indicating that vagus-mediated gut signaling, activates the hippocampus. Further monosynaptic and multisynaptic virus-based tracing investigation revealed a “medial nucleus tractus solitarius-medial septum-dorsal hippocampus glutamatergic neurons” connection, suggesting the existence of “gut-vagus-brainstem-septum-hippocampus” pathway [58]. Two types of vagal sensory neurons have been found to target the nucleus of the solitary tract (NTS) [85]. Moreover, various brain regions have been identified to be connected with the gut via the vagus nerve. Following the injection of pathological *α*-syn preformed fibrils into the duodenal and pyloric muscularis layer, pathologic *α*-syn could spread to the dorsal motor nucleus (DMN), caudal portions of the hindbrain (including the locus coeruleus), BLA, dorsal raphe nucleus (DRN), and the substantia nigra pars compacta (SNC). In addition, this gut-to-brain spread could be prevented by truncal vagotomy and *α*-syn deficiency [86]. This study supports the idea that the vagus nerve directly mediates the communication from the gut to the brain.

In the NTS, cholecystokinin-containing neurons, activation of which reduces appetite, are responsive to nutritional state and send projections to the paraventricular nucleus of the hypothalamus (PVH) [87]. The PVH also projects directly to the NTS [88], thereby establishing a connection from the brain to gut linked by the vagus nerve [80, 89]. Thus, the central autonomic network integrates the vagus nerve mediated visceral information and regulates the hypothalamic-pituitary-adrenal (HPA) axis [90], which is implicated in stress-related disorders [91, 92]. Moreover, cholecystokinin-4 administration could alter anxiety-like behavior and the HPA axis hormones such as corticosterone in rats exposed to early life stress [93].

To sum up, the vagus nerve-mediated gut-brain pathways at least involve “gut-vagus-NTS-septum-hippocampus” and “gut-vagus-DMN-hindbrain/BLA/DRN/SNC” pathways. The “NTS-PVH” loop might be a potential connection that regulates the “up-down” and “down-up” transmission. These complex neural pathways involve various stress-related brain regions, within which GABA signals play a crucial role. Alongside the gut-vagus-brain pathway, gut-associated factors, including cholecystokinin, NPY, and vasoactive intestinal peptide, act as modulators of GABA signaling, so as to regulate stress. Thus, we further discuss the crosstalk between the GABAergic system and the vagus mediated gut-brain pathway, especially the link with the hippocampus, amygdala, and hypothalamus, as well as related neural network.

### 3.2. The Crosstalk between the GABAergic System and the Vagus Nerve-Mediated Gut-Brain Pathways

The hippocampus is an important brain structure involved in various neural circuits and functions. Exposure to chronic stress has been shown to be accompanied by rising GABA levels in the dorsal hippocampus [94]. However, different stressors may cause distinct changes in hippocampal extracellular GABA levels; for instance, a novel environment increases GABA whereas forced swimming reduces GABA [7]. Interestingly, chronic stress affects specific GABAergic neuronal subpopulations in the hippocampus, including parvalbumin, calretinin, NPY, and somatostatin neurons, but not cholecystokinin and calbindin interneurons [8, 36]. The hippocampus receives inputs from the septum [95] and generates theta oscillations linked to multiple processes, including affect and locomotion [96, 97]. The septum receives inputs from the median raphe nucleus, in which inhibition of the GABAergic pathway affects theta oscillations and decreases anxiety [98]. Both the lateral and medial septum GABAA receptor signal can influence the hippocampal theta frequency, and the GABAA receptor agonist muscimol infused in the dorsal lateral septum reduces anxiety-like behavior [99]. Infusion of the GABAB receptor agonist baclofen into the lateral septum reduces stress-induced anorectic effect while increases sucrose intake [100]. It has been shown that early-life stress reduces GAD67 in the lateral septum [101]. These results suggest that the lateral septum GABAergic system is related to stress and food intake. Moreover, somatostatin interneurons in the dorsal lateral septum receive inputs from hippocampal CA3 directly [102], thereby forming a feedback loop between the hippocampus and the septum. The medial septum sends both GABAergic and glutamatergic outputs to the lateral habenula, which affects the aversion [103]. In addition, somatostatin interneurons in the hippocampus can be selectively inhibited by GABAergic neurons from the nucleus incertus, modulating of which can shift the hippocampal network state and modify fear [31]. Overall, the median raphe nucleus projects to the septum, which projects to the hippocampus and lateral habenula, and the nucleus incertus projects to the hippocampus, thus forming a complex neural network associated with stress.

Amygdala is associated with stress and fear regulation [104–107]. In patients with schizophrenia and bipolar disorder, somatostatin positive neurons decreased in the amygdala [42]. Selective activation of NPY neurons in the central amygdala (CeA) leads to increased food intake and decreased energy expenditure under stress [67]. GABAergic serotonin receptor 2a-expressing neurons in the CeA modulate food consumption [108]. Furthermore, BLA to CeA neural circuit also mediates appetitive behaviors [109]. Thus, the BLA-CeA microcircuit within the amygdala plays a potential role in regulating stress and stress-induced appetitive behaviors. In the BLA, 5-HT3aR positive GABAergic neurons are found, and the main coexpressing marker is cholecystokinin, very few express calretinin, vasoactive intestinal peptide, or parvalbumin, and none expresses somatostatin or calbindin [110]. Another study also shows that vasoactive intestinal peptide interneurons are found in the mouse BLA [111]. Dopamine in the BLA selectively suppressed GABAergic transmission from parvalbumin interneurons to principal neurons but not to interneurons [112]. Activation of BLA NPY2-receptors reduces tonic GABA release onto BLA principal neurons and increases anxiety [30]. Selective activation of the BLA-mPFC input provides a safety-signaling mechanism whereby the mPFC taps into the microcircuitry of the amygdala to reduce fear [113]. In general, BLA neurons project to the CeA and mPFC, and the GABA pathways within these circuits are implicated in stress regulation through multiple mechanisms.

The mPFC is an important brain region involved in the emotional memories. By using a rat model for depression, researchers examined the effect of stress on GABAergic system changes in the mPFC [114]. Nine weeks of chronic mild stress exposure has been shown to decrease the amount of cholecystokinin, calretinin, and parvalbumin-positive GABAergic neurons in the mPFC. In contrast, NPY-positive neurons are increased in the entire mPFC in stress-resilient rats. Moreover, the object-place paired-associate learning is impaired in stress-susceptible rats, suggesting that fronto-limbic GABAergic dysfunctions may contribute to emotional changes in depression [114]. In addition, chronic stress increases presynaptic GABA release, which is accompanied by increased inhibition onto prefrontal glutamatergic output neurons, leading to a reduced effect on modulating stress-related behavior [115]. The frontal cortex subregion cingulate projects to the primary visual cortex and affects visual discrimination. These long-range projections induce synaptic disinhibition of pyramidal neurons through local GABAergic neurons microcircuit, including vasoactive intestinal peptide, somatostatin, and parvalbumin-positive GABAergic interneurons [116].

The hypothalamus is a component of the HPA axis. Neurons in the hypothalamus subarea PVH produce corticotropin-releasing hormone (CRH) involved in endocrine stress response. GABAergic neurons projecting to the PVH regulate the excitability of CRH neurons [117]. Following adrenalectomy in rats, the synthetic and secretory activities of CRH neurons are increased, and a higher number of GABA-CRH synaptic contacts are detected in the PVH [118], suggesting a connection between the GABAergic system and the HPA axis. Moreover, a population of CRH positive GABAergic long-range-projecting neurons in the extended amygdala innervates the ventral tegmental area, and the chronic lack of CRH from this type of neurons produces anxiety [119]. Therefore, the GABAergic system may regulate anxiety-like behavior through the HPA axis and related networks. As described previously, there is a “NTS-PVH” loop linking to the “gut-vagus-NTS-septum-hippocampus” pathway. Intraperitoneal injection of cholecystokinin-8S increases the amount of activated neurons in the NTS and PVH [120], while activating the NTS cholecystokinin axon terminals within the PVH affects appetite [87, 121], suggesting the effects on the gut pathway. The vagus nerve stimulation reduces the CRH/adrenocorticotropic hormone responses in the depressed subjects [122], suggesting the potential connection between the vagus and the HPA axis. Collectively, the gut and vagus pathways are related to the HPA axis, at least in part, through the “NTS-PVH”.

### 3.3. Gut Microbiome in the GABAergic System and Vagal Communication

Growing evidence has shown that gut microbiome is involved in regulating stress-related behaviors and brain functions. Stress-associated anxiety- and depression-related behaviors are prevented by treatment with *Lactobacillus paracasei* Lpc-37, *Lactobacillus plantarum* LP12407, *Lactobacillus plantarum* LP12418, and *Lactobacillus plantarum* LP12151 [123]. *Lactobacillus plantarum* LP12418 can normalize the stress-induced reduction in adrenocorticotropic hormone [123]. Following probiotic bacterium *Lactobacillus rhamnosus* (JB-1) treatment, GABA levels are increased in the brain [124], and expression of GABA(B1*β*) and GABA(A*α*2) is changed in several brain regions, including the hippocampus (lower GABA(B1*β*), higher GABA(A*α*2)), amygdala (lower GABA(B1*β*) and GABA(A*α*2)), and prefrontal cortex (lower GABA(A*α*2)) [14]. One possible reason might be because many strains of Lactobacillus and Bifidobacterium are able to produce large quantities of GABA and activate GABA producing pathways [125, 126]. Moreover, *Lactobacillus rhamnosus* (JB-1) decreases stress-induced corticosterone and anxiety- and depression-related behavior, while no effects are found in vagotomized mice [14]. Similarly, *Lactobacillus plantarum* LP12418 also changes the expression of GABA(A*α*2) and GABA(B1*β*) in the prefrontal cortex [123]. *Lactobacillus casei* strain Shirota not only suppresses stress-induced increases in glucocorticoids both in subjects and in rats but also stimulates vagal afferent activity and suppresses stress-induced activation of CRF cells in the PVH [127]. However, the effects of *Lactobacillus rhamnosus* (JB-1) were still unsatisfying in modifying stress-related measures and HPA response in male subjects in a clinical trial [128]. Overall, gut microbiome may play an important role in regulating stress-related behavior through the GABAergic system and the gut-vagus-brain pathway.

## 4. Conclusion and Future Perspectives

**Figure 1:**
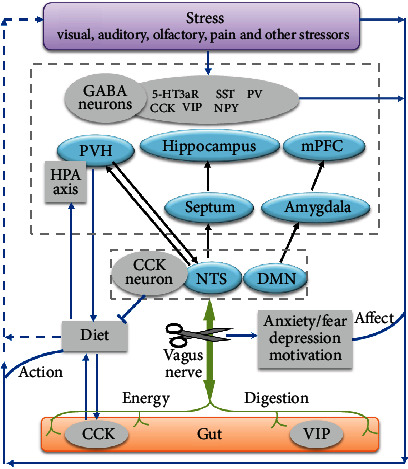
Potential role of crosstalk between the GABAergic system and the gut-vagus-brain pathway in stress. GABA, *γ*-aminobutyric acid; 5-HT3aR, serotonin receptor 5-HT3a; CCK, cholecystokinin; VIP, vasoactive intestinal peptide; SST, somatostatin; NPY, neuropeptide Y; PV, parvalbumin; mPFC, medial prefrontal cortex; PVH, paraventricular nucleus of the hypothalamus; NTS, nucleus of the solitary tract; DMN, dorsal motor nucleus; HPA, hypothalamic-pituitary-adrenal.

Stress can cause various mental changes and reactions, which may be attributed to multiple changes in the body, including the brain structure and related circuitry pathway. Several gut-related modulators, such as cholecystokinin, NPY, and vasoactive intestinal peptide, not only express in the digestive system but also exist in the CNS and colocalize with GABAergic neurons. In addition to regulating the diet, they are also implicated in stress, which involves various brain structures. Stress may alter gut-associated behavior, such as increased or decreased food intake [129, 130], which can further affect the gut hormone release [131]. The vagus nerve connects the gut with the brain bidirectionally, thereby establishing a gut-vagus-brain pathway. Thus, the crosstalk between the GABAergic system and the gut-vagus-brain pathway may play a potential role in stress (Figure 1).

Given that specific neuron types can be manipulated with chemogenetic and optogenetic approaches, the roles of different GABAergic neuron subgroups or relative cellular and molecular signals in stress can be further investigated. This may help find out more potential therapeutic targets for the treatment of stress-related CNS disorders, such as PTSD. It has been known that individuals with low plasma GABA levels are more susceptible to PTSD [132], which is commonly accompanied with functional gastrointestinal disorders [133]. Recent evidence indicates that gut microbiome is associated with stress-induced behaviors [134, 135]. Vagus nerve stimulation may improve PTSD-like symptoms [136]. Therefore, manipulation of the gut-vagus-brain pathway may have therapeutic potential for treating PTSD. However, the complex neuronal markers may lead to various functions of each GABAergic neuron subset in different brain regions or even in different subareas of the same region. In addition, the complicated connections between the GABAergic system and the gut-vagus-brain pathway may play a potential role in regulating stress. Further studies are needed to increase the target and region selectivity, which appears to be a challenge to the development of novel drugs or approaches for the treatment of stress-induced CNS disorders.
